# Eosinophilic Granuloma Involving the Femoral Neck

**DOI:** 10.1155/2013/809605

**Published:** 2013-05-23

**Authors:** Hoari Krishnan, Taek Rim Yoon, Kyung Soon Park, Je Hyoung Yeo

**Affiliations:** ^1^Department of Orthopaedic Surgery, Chonnam National University Hwasun Hospital, Chonnam National University Medical School, Jeonnam 519-809, Republic of Korea; ^2^Center for Joint Disease, Chonnam National University Hwasun Hospital, 322 Seoyong-ro, Hwasun-eup, Hwasun-gun, Jeonnam 519-809, Republic of Korea

## Abstract

We present a very rare case of eosinophilic granuloma involving the neck of femur in a 17-year-old male. The patient was treated with curettage and autologous iliac crest bone grafting with prophylactic screw fixation, and the diagnosis was confirmed by histopathological examination. Currently, it has been 14 years since the first surgery and the patient is well with no recurrence or relapse, and the osteolytic lesion has disappeared.

## 1. Introduction

It has been almost 7 decades since the term eosinophilic granuloma (EG) was first used by Lichtenstein and Jaffe [1]. They described the solitary EG of the femur in 1940. Two years later, Green and Farber [2] reported 10 cases of eosinophilic granuloma, and some had multiple lesions. In 1953, Lichtenstein [3] proposed the term histiocytosis X to describe three types of reticulosis: EG is the localized form, Lettere-Siwe's disease (LSD) is the acute form and Hand-Schüller-Christian disease (HSC) the chronic form. EG may also have lung or other visceral involvement in addition to bone lesions. Similarly, Schajowicz and Slullitel [4] concluded that EG, LSD, and HSC are of a single entity with similar pathological disorders. 

EG of bone is a disease of children and young adults. According to Teplik and Broder [5], only 5% occur in persons more than 30 years old, 75% of the lesions are solitary, and 59% of the lesions involve the skull; rib and femoral diaphysis are common sites for eosinophilic granuloma, but it is rarely found in the femoral neck. Kelley and Mcmillan [6] reported 9 cases of eosinophilic granuloma of bone in which 2 were in the femoral shaft. There has been only one case in the femoral neck described by McCullough [7], a healing lesion of femoral neck after biopsy and bone grafting. 

Now we report an extremely rare case of eosinophilic granuloma involving the femoral neck with prophylactic pinning with cannulated cancellous screws. The patient was informed that data concerning this case will be used for journal publication, and he consented.

## 2. Case Report

A 17-year-old male presented in June 1995 with left hip pain for 2-month duration. The patient was a high school student, and he had this pain on and off which was tolerable. At that time, he was active with his high school extracurricular activities. But he went to see his general practitioner as the pain was disturbing his school activities for 1 month. He took oral analgesia for the pain, and he came to our hospital as the pain worsened the last 10 days. There were no constitutional symptoms and no history of trauma. 

**Figure 1:**
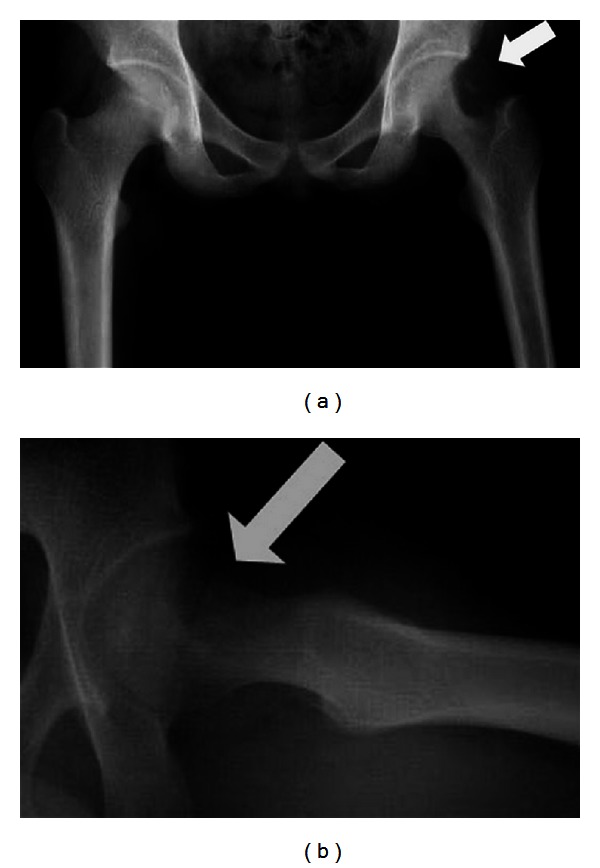
Radiograph showing (a) osteolytic lesion localized to superior aspect of left neck of femur with scalloping medullary margin and (b) the lesion over the anterior aspect of neck extending to head with intact anterior cortex (arrow).

**Figure 2:**
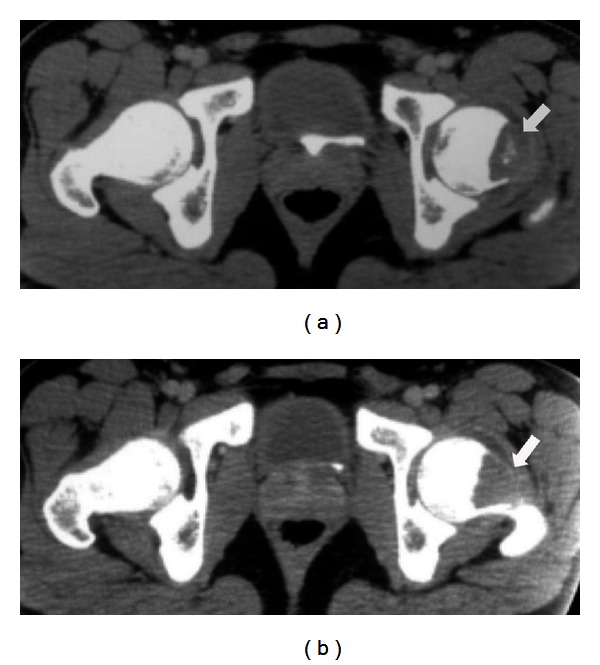
Axial CT scan of the pelvis showing (a) involvement of anterior aspect of neck of left femur with anterior cortical destruction and (b) the same lesion extending into the head of the left femur.

**Figure 3:**
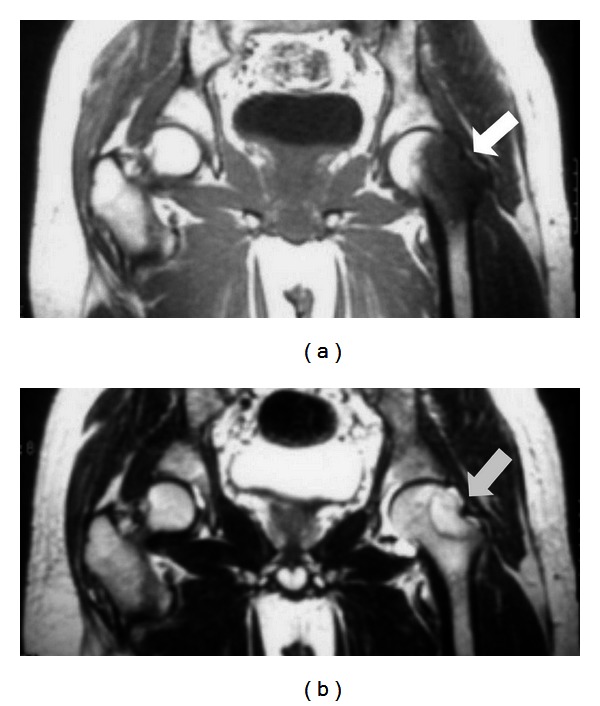
Coronal MRI pelvis showing (a) hypointense signal area in the marrow on T1-weighted image and (b) hyperintense signal area in the marrow on T2-weighted image, and there is no obvious extension of lesion into surrounding soft tissue.

On physical examination, the range of motion of the left hip was reduced due to pain. There was no lymphadenopathy or neurovascular deficit. The plain radiograph showed osteolytic lesion over the superior aspect neck of left femur with scalloping of medullary canal (Figure 1). CT scan reported a 4 cm destruction involving the metaphysis and neck of left femur with calcification and joint effusion and no sequestrum (Figure 2). MRI findings revealed a 4 cm well-circumscribed eccentric mass at neck of femur with abnormal marrow signals (Figure 3). The erythrocyte sedimentation rate (ESR) was slightly elevated (40 mm for the 1st hour), and the C-reactive protein (CRP) and total white cell count level were within normal range. Based on the history, clinical examination, and imaging, a diagnosis of benign bone tumor involving the neck of left femur was made.

**Figure 4:**
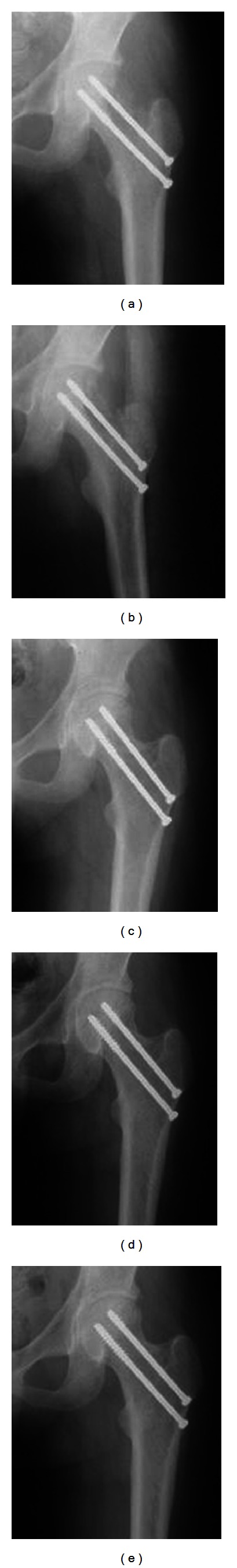
Radiograph showing from left to right the postoperative progress of the disease till 10-year followup. (a) The radiograph after curettage with bone grafting and screw fixation. (b) Six month followup, (c) 1-year followup, (d) 5-year followup and (e) 10-year followup. The subsequent radiograph shows the gradual disappearance of the osteolytic lesion, and 10 years after the surgery, the lesion has disappeared with new bony trabeculae formation.

 On anterior exploration of left hip joint, the capsule was intact; we opened up the capsule to reach the lesion. There was friable bone tissue of 4 × 3 cm over the anterior cortex of neck of femur which was breached, but the other cortices were intact. Curettage and autologous iliac crest bone grafting was carried out, as the frozen section confirmed the diagnosis of EG. Two cannulated screws were used to pin the neck of left femur prophylactically against pathological fracture. The histopathologic examination revealed a mixed cellular infiltrate consisting of eosinophilic polymorphs with plasma cells and Langerhans histiocytes. He had an uneventful postoperative period and was on non-weight-bearing crutches for 3 months. On the 4th month postoperatively the lesion showed a sign of healing, and partial weight bearing was commenced. He went back to high school after 6 months when he was on full weight bearing without any walking aid (see Figure 4).

**Figure 5:**
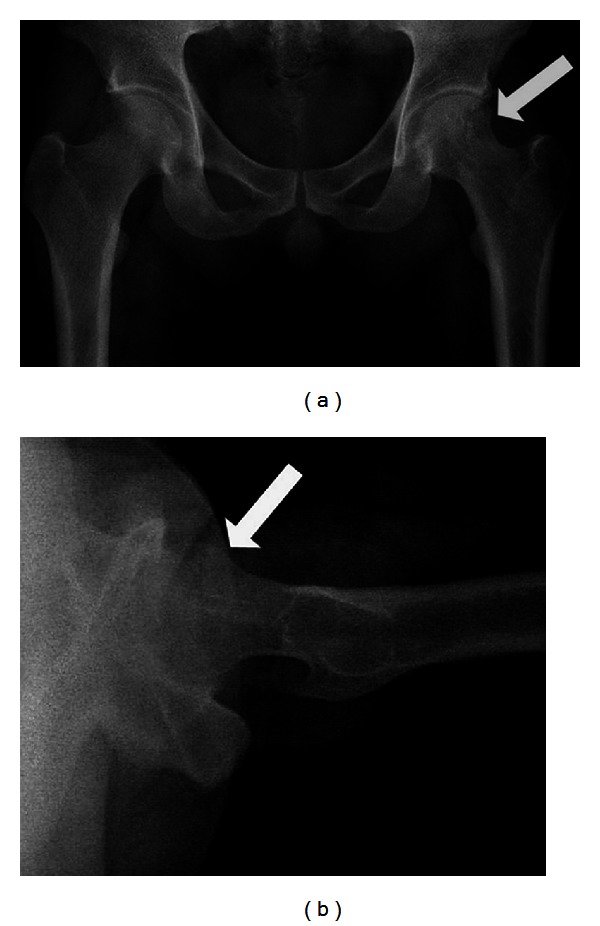
Radiograph taken 14 years after the initial surgery, showing the left neck of femur without the screws, and new trabeculae has formed over the left femoral neck with complete obliteration of the lesion.

 Now, it has been 14 years since the surgery and his EG of neck of femur has healed (Figure 5). He has no symptoms of recurrence or fracture, and the radiograph shows obliteration of the lesion with good trabeculae formation of the femoral neck which is visible clearly as the femoral neck screws have been removed.

## 3. Discussion

The prognosis is uncertain in EG, and spontaneous resolution may occur [7]. McCullough [7] in his series of 43 patients demonstrated that 36 patients had a solitary lesion and 7 patients presented with multiple bone lesions. Out of the solitary lesion patients, only 31 healed with no adverse sequelae and the remaining 5 patients developed polyostotic disease. Although EG is a destructive lesion of bone, but most solitary lesions healed without complication [7]. This was also described by Nauert et al. [8]: EG is a benign lesion that on occasion heals spontaneously.

 Many authors have reported various types of treatment for this disease. The choice of treatment depends on the extent of the disease and location [9]. As from the literature, we have options of observation, radiotherapy, chemotherapy, local steroid injection, and surgery. In 1983, Ruff et al. [10] described a case of eosinophilic granuloma in the humerus of a three-and-half-year-old boy. After open biopsy and local steroid, the lesion healed in 10 months. More recently, injection with methylprednisolone has shown favorable results. Although the mechanism of intralesional injection of methylprednisolone has not been defined but by the usage of percutaneous needle biopsy to diagnose EG and to administer methylprednisolone, this enables the patient to avoid open surgery and to give prompt relieve of pain [11]. 

Plasschaert et al. [12] noticed spontaneous recovery of the lesion in skeletally immature patients after biopsy with and without bone grafting. A feature of prognostic significance was an increase in the size of the presenting skeletal lesion following biopsy with curettage, and it would seem that in the majority of patients further lesions will not appear once existing lesions start to heal [5]. Although Cheyne [13] has described one patient who developed further bone lesions after the presenting lesion had healed our patient started to show healing by one year, and there were no other lesions since then.

The role of radiotherapy (RT) is still controversial. Kelley and Mcmillan [6] recommended RT for multiple lesions or lesions which do not respond to curettage. However, in contrast, McCullough [7] noticed in some patients that RT did not control local bone destruction and did not prevent subsequent new bony lesions. 

In most of cases, curettage is the treatment of choice, especially in easily accessible solitary lesions like in long bones [4] and in solitary lesion [9]. Similarly, in our case, treatment was carried out for symptomatic pain relief, where curettage with autologous iliac crest bone grafting was performed as the defect was more than 4 × 3 cm, and to decrease the risk of pathological fracture [14], screw fixation was done. 

As the treatment regimens varied with different physicians [15], hence there is no general consensus for treatment of EG, and a further prospective study of different surgical approaches in age-matched groups of patients is needed.
